# Advances in Radiomics Research for Endometrial Cancer: A Comprehensive Review

**DOI:** 10.7150/jca.89347

**Published:** 2023-10-24

**Authors:** Wenxiu Guo, Tong Wang, Binglin Lv, Jie Jiang, Yao Liu, Peng Zhao

**Affiliations:** 1 Department of Radiology, Shandong Provincial Hospital Affiliated to Shandong First Medical University, Jinan, Shandong Province, 250021, China.; 2 Department of Gynecology and Obstetrics, Qilu Hospital of Shandong University, 250012, Jinan, China.

**Keywords:** Endometrial Cancer, Radiomics, Pathological Features, Prognostic Analysis

## Abstract

Endometrial cancer (EC) is a common gynecologic malignancy, with a rising trend in related mortality rates. The assessment based on imaging examinations contributes to the preoperative staging and surgical management of EC. However, conventional imaging diagnosis has limitations such as low accuracy and subjectivity. Radiomics, utilizing advanced feature analysis from medical images, extracts more information, ultimately establishing associations between imaging features and disease phenotypes. In recent years, radiomic studies on EC have emerged, employing radiomic features combined with clinical characteristics to model and predict histopathological features, protein expression, and clinical prognosis. This article elaborates on the application of radiomics in EC research and discusses its implications.

## Introduction

Endometrial cancer (EC) stands as a prevalent gynecological malignancy, with an annual global incidence of approximately 417,000 newly diagnosed cases 1. In the context of China, the year 2016 witnessed about 71,100 fresh EC cases and an estimated 17,100 fatalities 2. Despite a generally favorable prognosis for EC, the scenario becomes grim for patients grappling with advanced or recurrent stages, characterized by significantly diminished outcomes. Notably, the 5-year overall survival rates for stages IVA and IVB are restricted to a mere 17% and 15%, respectively 3. Moreover, a concerning upward trajectory in EC-related mortality has been observed 4, with the survival rate for females afflicted with uterine malignancies showing no enhancement over the preceding four years 5. The prognosis of EC is intricately influenced by a spectrum of factors, fostering the development of risk assessment frameworks grounded in FIGO staging, grading, histological variations, lymphovascular space invasion (LVSI), and molecular subtypes 6.

In the realm of EC management, surgical pathological staging retains a pivotal role, complemented by imaging examinations that facilitate refined preoperative staging and surgical planning. Ultrasonography emerges as the primary tool for early-stage disease screening, while magnetic resonance imaging (MRI) serves to delineate myometrial and cervical stromal invasion, thereby enhancing preoperative staging accuracy. Although computed tomography (CT) offers limited sensitivity (83%) and specificity (42%) in pinpointing myometrial and cervical stromal invasion, it finds utility in the later stages, particularly for the detection of extrauterine lesions. Positron emission tomography/computed tomography (PET/CT), on the other hand, proves instrumental in identifying extrapelvic involvement, boasting high specificity in the detection of lymph node metastasis (94%) and EC recurrence (90-100%) 7. Moreover, innovative approaches such as three-dimensional transvaginal ultrasound (3D-TVS) are gaining traction in the preoperative evaluation of EC. Despite the extensive deployment of these imaging modalities, conventional diagnostic strategies encounter certain limitations. A pertinent study encompassing 91 EC patients disclosed that a mere 47.2% of MRI evaluations accurately forecasted the disease stage 8, with the efficacy of ultrasonography being potentially undermined by elements like leiomyomas impacting the myometrial layers 9. These hurdles necessitate a profound investigation into the potentialities of medical imaging data.

**Figure 1:**
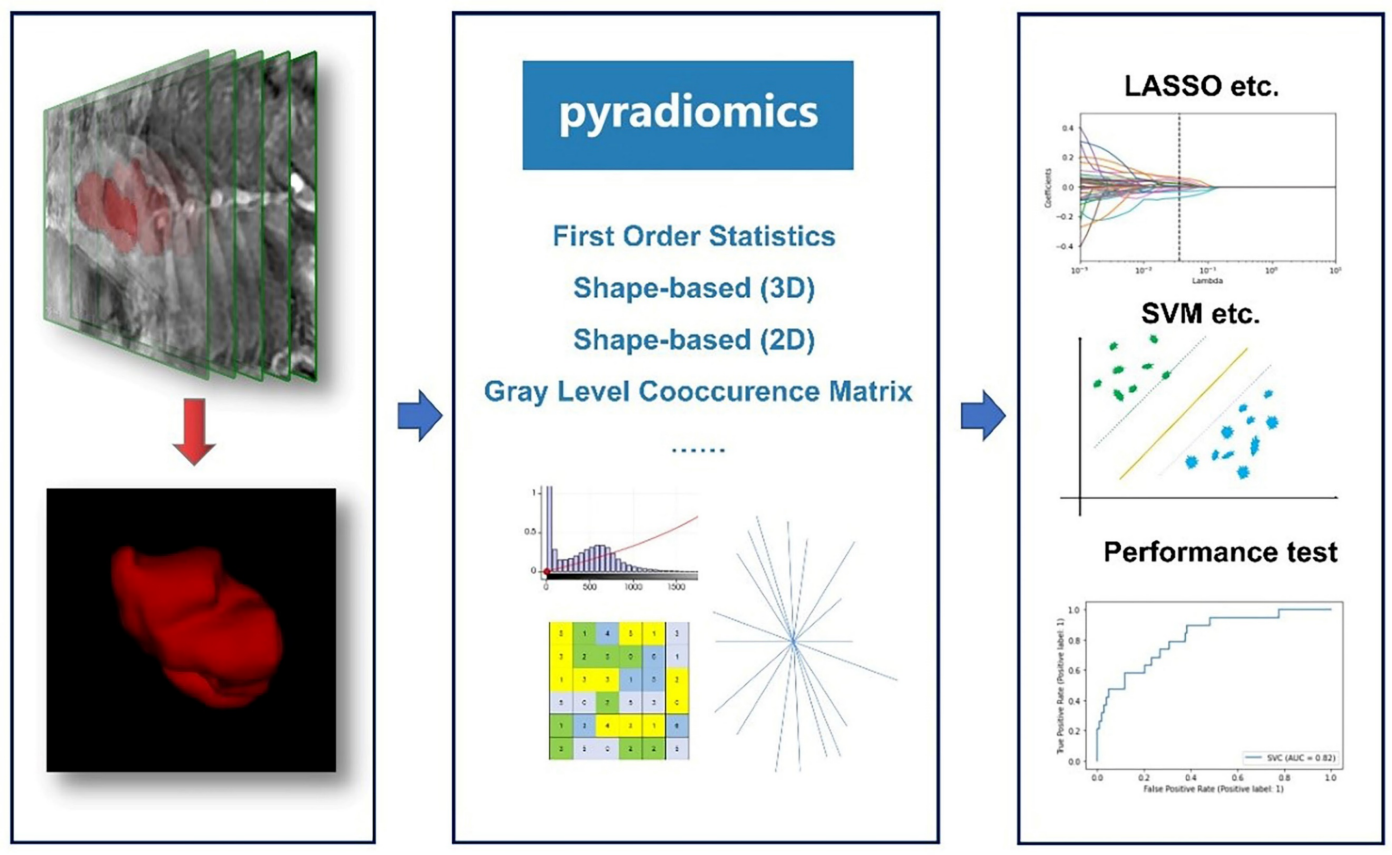
General framework showing the main steps of the radiomics. The general workflow of radiomics includes acquiring high-quality imaging data, delineating Regions of Interest (ROIs) using various relevant software, extracting features based on PyRadiomics, filtering features using different statistical methods, and finally constructing and validating the predictive models.

**Figure 2:**
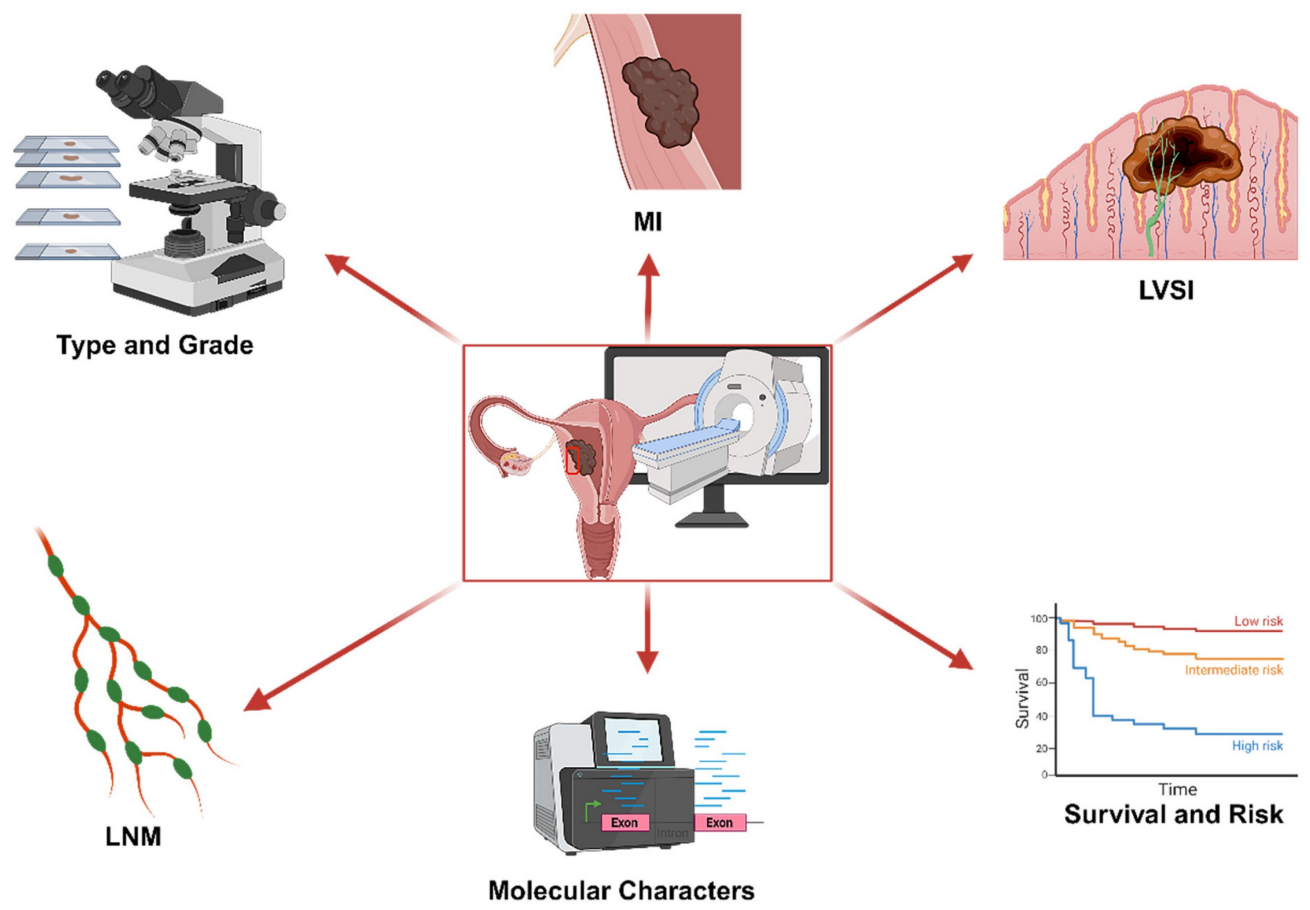
The utilization of radiomics in EC.

Introduced in 2012, the field of radiomics, alternatively termed radiogenomics, aspires to unlock a wealth of information encapsulated in medical images through sophisticated feature analysis 10. This domain encompasses procedures such as image segmentation, feature extraction, and selection, coupled with analytical techniques to forge associations between imaging attributes and disease phenotypes. In a significant stride, Griethuysen et al. 11 unveiled the open-source platform PyRadiomics, accessible at [https://pyradiomics.readthedocs.io/en/latest/] (https://pyradiomics.readthedocs.io/en/latest/), which heralded the standardization of algorithms and image processing protocols, thereby amplifying the efficiency of research workflows. Figure 1 delineates the overarching framework of radiomics. In the ensuing years, the radiomics sector witnessed a surge in studies, inclusive of those centered on EC, leveraging primarily MRI, supplemented by CT and PET-CT, and to a lesser extent, ultrasound imagery. These investigations predominantly focus on modeling imaging characteristics and, to a certain degree, clinical parameters, with the aim of predicting histopathological attributes, protein expression profiles, and clinical prognosis. Figure 2 illustrates the integration of radiomics in EC research, setting the stage for an in-depth exploration of the nuances of EC radiomics in the subsequent discussion.

## 1. Evaluation of Myometrial Invasion Depth in Initial Stages of Endometrial Carcinoma

Myometrial invasion (MI) is a critical parameter that significantly influences surgical planning and prognosis in EC, being closely related to lymph node metastasis 12. Currently, MRI stands as the principal tool for preoperative MI evaluation in EC 13. However, the task of distinguishing between superficial and deep MI based solely on MRI imagery can be intricate, often necessitating the discerning judgment of physicians for accurate staging 12.

Recent studies have ventured into the potential of utilizing multiparametric MRI radiomics along with machine learning classifiers in the preoperative assessment of EC. A study by Otani, Himoto et al. 14 scrutinized the efficacy of these classifiers, noting an average Area Under the Receiver Operating Characteristic Curve (AUROC) of 0.83 for the Deep Myometrial Invasion (DMI) classifier, a score comparable to the performance of experienced radiologists. In a similar vein, the research conducted by Han's team 15 encompassed 163 EC patients and focused on extracting 24 radiomics features from T2-weighted imaging (T2WI) and diffusion-weighted imaging (DWI), achieving an AUROC of 0.85 in the validation set, a score not significantly different from subjective diagnoses (P>0.05). Furthermore, the team led by Stanzione 16 managed to attain an AUROC of 0.92 using a radiomics feature classifier, enhancing the accuracy of radiologists from 82% to a perfect 100%. Another noteworthy study by Rodríguez-Ortega, Alegre et al. 17 integrated radiomics features with parameters such as the apparent diffusion coefficient (ADC) and time to peak in their classifier model, achieving an optimal performance in identifying outcomes with MI≥50% (accuracy 86.1%, AUROC 0.871).

In addition, the development of radiomics-based nomograms has emerged as a promising tool in MI assessment in EC. A study by Zhao et al. 18 amalgamated clinical and conventional imaging indicators with radiomics features to construct a nomogram, registering an AUROC of 0.871 in the validation set, closely aligning with ideal benchmarks. Similarly, research by Wang et al. 19 combined clinical data with radiomics features, utilizing a nomogram to proficiently predict DMI, with an AUROC of 0.883. This tool notably enhanced the diagnostic accuracy of radiologists, elevating it from 79.0% and 80.2% to 90.1% and 92.5%, respectively. In a parallel development, Yan, Ma et al. 20 developed a nomogram for predicting ovarian preservation in early-stage EC, achieving an AUROC of 0.96, thereby surpassing the performance of radiologists (0.80 and 0.86). To address the challenge of detecting non-visible EC in MRI, Jiang's team 21 developed two radiomics models for distinguishing non-visible EC and MI, both demonstrating excellent performance with average AUROCs of 0.896 and 0.844, respectively. The current body of research indicates that classifiers based on radiomics features, either standalone or in conjunction with selected clinical and traditional imaging features, hold high diagnostic efficacy, potentially matching or exceeding the capabilities of seasoned radiologists, thereby enhancing diagnostic precision.

## 2. Prediction of Tissue Typing and Histological Grading

The conventional categorization of EC hinges on histological attributes, bifurcating it into estrogen-dependent (Type I) EC, encompassing Grade 1 and Grade 2 endometrioid adenocarcinomas, and non-estrogen-dependent (Type II) EC, which includes Grade 3 endometrioid adenocarcinoma along with non-endometrioid variants such as serous carcinoma, clear cell carcinoma, undifferentiated carcinoma, and carcinosarcoma 22. Despite the gradual shift towards molecular classification in the EC domain, histological traits and grading retain their significance as pivotal evaluation parameters, as underscored by the recent 2022 ESMO guidelines 6. Presently, radiomics research predominantly leverages MRI technology, concentrating on the differentiation of early-stage EC from benign conditions or precancerous lesions, and distinguishing among diverse histological EC variants.

### 2.1 Histological Variants

Recent research spearheaded by Bi et al. 23 in a multicentric study encompassing 371 patients, meticulously extracted radiomics features from T2WI, DWI, ADC maps, and late contrast-enhanced T1-weighted imaging (LCE-T1WI). Utilizing logistic regression, they formulated a radiomics model adept at distinguishing IA-stage EC from benign endometrial lesions, achieving an accuracy of 0.802 and exhibiting high AUROC values in both internal and external validations (average 0.854). Concurrently, another multicentric study conducted by Chen, Wang et al. 24 employed five selected radiomics features derived from ADC, T2WI, and DWI to develop a linear kernel support vector machine model, which demonstrated a remarkable average AUROC of up to 0.983 in distinguishing EC from benign conditions such as endometrial polyps. Moreover, Zhang et al. 25 crafted a radiomics-clinical model, integrating radiomics features with clinical indicators such as uterine endometrial thickness exceeding 11mm and nulliparous status, to differentiate endometrial carcinoma from atypical endometrial hyperplasia, attaining an AUROC of 0.942 in the validation set. Xie et al. 26 led a study comparing MRI features and radiomics features in distinguishing uterine sarcoma from atypical leiomyoma, where the radiomics model slightly outperformed radiologists with an AUROC of 0.830 as opposed to 0.752. Furthermore, radiomics-based nomograms have been effectively utilized to differentiate between Type I and Type II EC, with a retrospective study involving 875 EC patients achieving AUROCs of 0.93 (training set) and 0.91 (testing set) 27.

### 2.2 Histological Grading

Nevertheless, the current landscape of research exhibits a discernible gap in substantial evidence affirming the superiority of imaging techniques in predicting histological grading. Initiatives undertaken by various research groups 14, 28 aimed at forecasting histological grading through MRI-based radiomics classifiers have yielded average AUROC values of 0.77 and 0.64, respectively. These figures fall short of the benchmark set by the tumor short axis measurement of ≥20mm (AUROC=0.86), indicating a room for enhancement in diagnostic performance.

## 3. Prognostication of Lymphovascular Space Invasion (LVSI) Status

LVSI, characterized by the infiltration of tumor cells within the lymphatic or vascular channels of the uterine myometrium, serves as a pivotal prognostic marker and stands as an autonomous risk determinant for lymph node metastasis and adverse prognosis 29, 30. The preoperative ascertainment of LVSI status is instrumental in clinical stewardship, particularly influencing surgical strategizing.

In the context of EC, the prognostication of LVSI via radiomics as a standalone approach appears to encounter certain impediments. Research endeavors spearheaded by Celli et al. 31 and Bereby-Kahane et al. 28, among others, have ventured into the development of radiomics models predicated on ADC and T2WI to prognosticate LVSI status. However, these efforts have culminated in AUROC values merely hovering around 0.59, thereby denoting a confined efficacy. Conversely, the integration of parameters derived from diverse modalities markedly amplifies the predictive prowess. For instance, Luo et al. 32 orchestrated a multi-modal nomogram, registering AUROC values of 0.820 and 0.807 in the training and testing sets respectively. Furthermore, Long et al. 33 augmented the model with computer vision attributes, thereby elevating the average AUROC from 0.77 to a notable 0.87. In a parallel multicentric investigation conducted by Liu, Yan et al. 34, a radiomics nomogram, enriched with age and carbohydrate antigen 125 (Ca125) parameters, attained AUROCs of 0.89 and 0.85 in the training and testing sets respectively, underscoring its efficacy in LVSI status prognostication. Hence, it is discernible that the adoption of a multi-modal parameter framework manifests as a superior strategy in enhancing the predictive accuracy for LVSI status in EC.

## 4. Prognostication of Lymph Node Metastasis (LNM)

Lymph node metastasis (LNM) is recognized as a significant prognostic determinant in EC 35. Historically, pelvic and para-aortic lymphadenectomy have constituted the preliminary surgical interventions, with the risk of LNM oscillating between 5% and 40% 36. Nonetheless, LNM can precipitate lymphedema in upwards of 30% of patients, alongside heightened bleeding risks 37. Despite the lack of evidence showcasing enhanced outcomes associated with systematic pelvic lymphadenectomy (LNE) in EC from prospective randomized trials 38, 39, lymphadenectomy remains pivotal in steering adjuvant treatment strategies and furnishing prognostic insights, necessitating the evaluation and excision of suspect lymph nodes.

The diagnostic prowess of MRI in discerning LNM in EC patients is somewhat limited, exhibiting a sensitivity of 43% and specificity of 73%, and faltering in distinguishing between inflammatory enlargement and metastatic nodes 40-42. Conversely, while PET/CT manifests high specificity (94-99%), its sensitivity (33-72%) in detecting LNM in EC patients is compromised, particularly in identifying nodes with dimensions less than 5mm 7, 43, 44. The progression of functional MRI in this domain is somewhat restrained, albeit radiomics has facilitated a degree of amelioration.

In the pursuit of prognosticating LNM in lymph nodes of standard size, Xu and team 45 orchestrated a predictive model leveraging dynamic contrast-enhanced MRI (DCE MRI) and clinical parameters. This comprehensive model, encapsulating radiomics attributes, lymph node dimensions, and Ca125, demonstrated commendable diagnostic efficacy, registering AUROCs of 0.892 and 0.883 in the training and testing sets respectively. Parallel studies 46, 47 recorded AUROCs of 0.940 and 0.85 respectively in LNM prognostication, markedly surpassing the average ADC model (0.54) and the standard for lymph node short-axis diameter (0.62). Notwithstanding, the inclusion of tumor grade in Yang's model imposes certain constraints. Liu et al. 48 formulated a radiomics nomogram integrating MRI-based radiomics attributes and Ca125, exhibiting a high predictive accuracy for LNM (average AUROC = 0.83). Moreover, a multicentric investigation 42 exclusively reliant on radiomics attributes showcased remarkable predictive performance for LNM across training and dual testing sets (AUROCs = 0.935, 0.909, 0.885), significantly outperforming manual radiologist evaluations. However, the incorporation of 37 features in this model incites debates regarding its efficiency and applicability.

The extraction, selection, and modeling of radiomics attributes can notably enhance the sensitivity of PET/CT in LNM prognostication. A research endeavor 44 amalgamated unique heterogeneity attributes derived from primary tumors with conventional visual detection, achieving sensitivities of 94% and 89% in two cohorts for LNM detection, a substantial improvement from the 50% and 33% attained through visual detection alone. Soydal et al. 49 identified critical radiomics attributes for LNM prognostication via texture features and data mining, attaining an accuracy rate of 0.8. Another group 50 computed 167 radiomics attributes within the tumor contour utilizing the Image Biomarker Standardization Initiative (IBSI) methodology, selecting 64 features with significant correlations to LNM, and ultimately pinpointing volume density as the paramount predictive attribute (AUROC = 0.77). The amalgamation of radiomics attributes with other multi-modal parameters can substantially augment the accuracy of LNM prognostication in EC.

## 5. Molecular Characteristics

The recent ESMO guidelines have ushered in a new era of risk assessment for EC, integrating molecular subtyping into the evaluation process 6. The POLE ultramutated (POLE mut) subtype is indicative of a promising prognosis, whereas p53 abnormalities (p53abn) denote a substantial advantage from adjuvant chemoradiotherapy, as opposed to radiotherapy in isolation 51. The deficiency in mismatch repair (MMRd) is intrinsically linked to Lynch syndrome, prompting numerous global organizations to advocate for universal MMRd testing in EC patients 52. This approach potentially facilitates the effective deployment of immune checkpoint inhibitors in MMRd patients 51. Furthermore, pinpointing potential target pathways such as PI3K-AKT or FBXW7-FGFR2 holds significance, especially in tumors with a grim prognosis 53. At present, the primary method for POLE mut testing is sequencing, while the identification of MMRd, p53abn, and significant pathway targets predominantly occurs through pathological tissue immunohistochemistry 52. However, these tests necessitate postoperative pathological specimens and entail substantial costs. Radiomics emerges as a beacon of hope, offering a non-invasive avenue for the preoperative prediction of molecular subtypes, prognosis, and treatment-related protein expression in EC. Models grounded in high-reliability radiomic classification have the potential to supersede traditional immunohistochemistry and sequencing methods, facilitating early-stage, precise treatment plan selection for patients, particularly before the availability of sequencing results 53.

In 2020, a pioneering study 54 embarked on an exploration to ascertain if radiomics attributes derived from contrast-enhanced computed tomography (CE-CT) could discern MMRd and/or high tumor mutation burden (TMB-H) in EC. The model, which amalgamated radiomics and clinical features, adeptly differentiated MMRd from copy-number low (CN-L) and copy-number high (CN-H) EC, registering an AUROC of 0.78. Moreover, it successfully segregated TMB-H from low tumor mutation burden (TMB-L), achieving an AUROC of 0.87, thereby affirming the supportive role of radiomics in molecular spectrum analysis. Another research initiative 55 detected disparities in the gray level co-occurrence matrix entropy (GLCM Entropy) within PET/CT radiomics features between Lynch syndrome and non-Lynch syndrome EC, with significant statistical results (p<0.001, AUROC=0.94 in the training set, 0.893 in the testing set). This study also unveiled correlations between GLCM Entropy variations and PD1 expression, based on RNA-seq genomic data in a separate cohort comprising 23 patients. Furthermore, a group formulated a radiomic prognostic index (RPI) grounded in MRI radiomics features, establishing a notable link between high RPI and adverse prognosis (p<0.001). This discovery spurred further investigation into mRNA expression disparities between high and low RPI groups, unveiling prognostically pertinent differentially expressed genes COMP and DMBT1. A comprehensive study encompassing 866 EC patients 56 identified correlations between radiomics features and specific transcriptional programs, marking a significant milestone in imaging genomics and furnishing fresh perspectives and benchmarks for ensuing EC radiomics research.

In the context of reproductive-aged women aspiring to retain fertility and grappling with atypical endometrial hyperplasia (AEH) or EC, a preference for conservative treatment primarily anchored in endocrine therapy is prevalent. Generally, individuals harboring POLE mutations exhibit a favorable prognosis, although the simultaneous presence of PTEN-negative/CTNNB1-positive expressions could amplify the risk of carcinogenesis or cancer progression. However, the current scientific landscape lacks conclusive evidence to delineate the molecular subtypes of endometrial cancer that would benefit maximally from conservative treatment 57. The inception of classification models within the cohort opting for conservative treatment for EC, integrating radiomic features with or without molecular subtyping, to promptly determine patient suitability for conservative treatment, emerges as a promising avenue for future research. In summation, while the exploration of this domain for EC is nascent, it harbors substantial potential and exploratory value.

## 6. Survival Analysis and Risk Stratification

The field of radiomics is witnessing a growing trend where research teams are venturing beyond the confines of single-index modeling predictions to apply radiomics directly in the survival analysis and risk stratification of EC. These models, which amalgamate predicted outcomes, are noted for their enhanced usability and clinical accessibility.

At present, the majority of radiomics models devised for EC risk stratification are grounded in MRI technology. However, the criteria utilized for risk stratification in EC are diverse. While some studies earmark high risk based on parameters like MI, LNM, and histological type I/II, others extend their criteria to include LVSI and histological grading. This absence of standardized criteria poses a challenge for clinical implementation, yet the insights garnered from these studies remain a valuable reference. A notable study conducted by Mainenti, Stanzione et al. 58 encompassed 133 patients from two different institutions and zeroed in on four radiomics features for their modeling, achieving an average AUROC of 0.715 in distinguishing high-risk from low-risk EC cases. In a similar vein, Zhang's group 59 merged MRI radiomics features with ADC to formulate a radiomics-based column chart model, facilitating the classification of EC types, grading, DMI, LVSI, and LNM, with AUROCs ranging between 0.746 and 0.959. Another multicentric study spearheaded by Yan, Li et al. 60 employed a column chart model to predict high-risk EC, registering promising AUROCs of 0.896, 0.877, and 0.919 across the training set and two testing sets. Lefebvre and colleagues 61 delineated high-risk EC based on factors such as DMI, LVSI, and advanced FIGO stage, with model AUROCs oscillating between 0.74 and 0.84. Moreover, a study by Chen et al. 62 centered on stage I EC, where their radiomics model surpassed models based on clinical and routine MRI features in predicting low-risk and intermediate-high-risk EC, boasting an AUROC of 0.946 as opposed to 0.756. In a recent endeavor, Moro, Albanese et al. 63 applied radiomics analysis to ultrasound imagery, crafting three distinct models (radiomics model, clinical ultrasound model, mixed model) for the prediction of high-risk EC. These models exhibited AUROCs of 0.80, 0.90, and 0.88 in the validation set for high-risk predictions, and 0.71, 0.85, and 0.80 for low-risk predictions. Despite demonstrating predictive prowess, radiomics applied to ultrasound images did not significantly outperform traditional models, with the subjective nature of ultrasound image acquisition serving as a limitation. As of now, there are no published studies on radiomics targeting the latest EC risk stratification guidelines.

Simultaneously, several studies have made strides in crafting prognostic prediction models by marrying radiomics features with clinical characteristics. A retrospective study encompassing 53 EC patients 64 integrated PET/CT radiomics features with certain clinical characteristics to formulate a model predicting disease progression, with the k-nearest neighbors (kNN) machine learning algorithm propelling it to the highest performance tier with an AUROC of 0.890. Liu and team 65 devised a column chart model to forecast 5-year progression-free survival (PFS), utilizing radiomics features extracted from a retrospective analysis of 202 EC patients, outclassing a basic clinical prediction model with AUROCs of 0.840 (training set) and 0.958 (testing set). Furthermore, Li et al. 66 selected three radiomics features from T2WI MRI and amalgamated them with two clinical variables to create a Cox proportional hazards (CPH) model for survival time prediction, which exhibited superior performance in testing compared to the clinical model, with an AUC of 0.727 against an AUROC of 0.624.

## Summary and Prospects

In recent times, the sphere of radiomics research pertaining to EC has undergone substantial growth, with a marked reliance on MRI as a primary tool. These studies are increasingly incorporating clinical data and other multimodal parameters to formulate predictive models that span various prognostic facets including histological classification, grade, MI, LVSI, LNM, and analyses of molecular or protein expressions. A significant shift has been observed from single-parameter predictions to more comprehensive analyses focusing on risk stratification and survival outcomes, demonstrating promising diagnostic efficacy.

However, the path to further progress and clinical implementation is fraught with challenges. A primary concern is the inherent subjectivity and variability in the extraction of radiomics features. Despite the widespread adoption of platforms like PyRadiomics for standardized algorithm definition and image processing, inconsistencies in feature definitions remain a hurdle. The lack of uniformity in the radiomics feature extraction process is exacerbated by variations in equipment and sampling parameters across different institutions, coupled with the subjective approach to lesion delineation. Moreover, the manual segmentation of tumor lesions not only introduces a high degree of subjectivity but also necessitates substantial manpower resources. Recent initiatives, as seen in the works of Hodneland, Dybvik et al. 67 and Kurata, Nishio et al. 68, have ventured into utilizing deep learning techniques, such as convolutional neural networks, to automate the segmentation of EC lesions on MRI, thereby enhancing the reliability of radiomics feature extraction. As the domain of artificial intelligence (AI) progresses, the mainstream trajectory seems to be leaning towards automated extraction through AI networks. However, the learning curve associated with radiomics research is steep, and the scarcity of user-friendly tools hampers its widespread adoption, standing in stark contrast to the accessibility found in traditional clinical research.

Moreover, fostering effective collaboration between radiology and clinical departments is of paramount importance. The existing gap in understanding between radiologists and the specific demands of clinical research, along with a limited grasp of the latest developments in specialized clinical research, sometimes culminates in diminished clinical relevance of certain radiomics studies.

As we forge ahead in the big data era, the focus is shifting towards the creation of automated radiomics feature extraction systems and multimodal predictive platforms grounded in AI networks, promising to be a dominant force in EC research. These systems, characterized by their non-invasive nature and precision, hold the potential to significantly influence early screening, differential diagnosis, preoperative staging, the choice of adjuvant therapy, and monitoring of disease progression in EC, heralding a new chapter in the fight against this disease.

## Abbreviations

EC: Endometrial cancer
LVSI: lymphovascular space invasion
MRI: Magnetic resonance imaging
CT: Computed tomography
PET/CT: Positron emission tomography/computed tomography
3D-TVS: three-dimensional transvaginal ultrasound
MI: Myometrial invasion
AUROC: the average Area Under the Receiver Operating Characteristic Curve
DMI: Deep Myometrial Invasion
T2WI: T2-weighted imaging
DWI: diffusion-weighted imaging
ADC: apparent diffusion coefficient
LCE-T1WI: late contrast-enhanced T1-weighted imaging
LNM: Lymph node metastasis
DCE MRI: dynamic contrast-enhanced MRI
IBSI: Image Biomarker Standardization Initiative
MMRd: Mismatch repair deficiency
CE-CT: contrast-enhanced computed tomography
TMB-H: high tumor mutation burden
CN-L: copy-number low
CN-H: copy-number high
TMB-L: low tumor mutation burden
GLCM Entropy: gray level co-occurrence matrix entropy
RPI: radiomic prognostic index
kNN: k-nearest neighbors
PFS: progression-free survival
CPH: Cox proportional hazards
